# NMDA and GABA Receptor-Mediated Plasticity Induced by 10-Hz Repetitive Transcranial Magnetic Stimulation

**DOI:** 10.21203/rs.3.rs-4630964/v1

**Published:** 2024-06-26

**Authors:** Jamie Kweon, Megan Vigne, Andrew M. Fukuda, Boyu Ren, Linda L. Carpenter, Joshua C. Brown

**Affiliations:** Brain Stimulation Mechanisms Laboratory, Neurotherapeutics, Division of Depression and Anxiety, McLean Hospital; Department of Psychiatry and Human Behavior, Alpert Medical School of Brown University, Butler Hospital; Brain Stimulation Mechanisms Laboratory, Neurotherapeutics, Division of Depression and Anxiety, McLean Hospital; Department of Psychiatry, Harvard Medical School; Department of Psychiatry and Human Behavior, Alpert Medical School of Brown University, Butler Hospital; Brain Stimulation Mechanisms Laboratory, Neurotherapeutics, Division of Depression and Anxiety, McLean Hospital

**Keywords:** rTMS, plasticity, NMDA, GABA

## Abstract

Although 10-Hz repetitive transcranial magnetic stimulation (rTMS) is an FDA-approved treatment for depression, we have yet to fully understand the mechanism through which rTMS induces therapeutic and durable changes in the brain. Two competing theories have emerged suggesting that 10-Hz rTMS induces N-methyl-D-aspartate receptor (NMDAR)-dependent long-term potentiation (LTP), or alternatively, removal of inhibitory gamma-aminobutyric acid receptors (GABARs). We examined these two proposed mechanisms of action in the human motor cortex in a double-blind, randomized, four-arm crossover study in healthy subjects. We tested motor-evoked potentials (MEPs) before and after 10-Hz rTMS in the presence of four drugs separated by 1-week each: placebo, NMDAR partial agonist d-cycloserine (DCS 100mg), DCS 100mg + NMDAR partial antagonist dextromethorphan (DMO 150mg; designed to “knock down” DCS-mediated facilitation), and GABAR agonist lorazepam (LZP 2.5mg). NMDAR agonism by DCS enhanced rTMS-induced cortical excitability more than placebo. This enhancement was blocked by combining DCS with NMDAR antagonist, DMO. If GABARs are removed by rTMS, GABAR agonism via LZP should lack its inhibitory effect yielding higher post/pre MEPs. However, MEPs were reduced after rTMS indicating stability of GABAR numbers. These data suggest that 10-Hz rTMS facilitation in the healthy motor cortex may enact change in the brain through NMDAR-mediated LTP-like mechanisms rather than through GABAergic reduction.

## Introduction

Repetitive transcranial magnetic stimulation (rTMS) has demonstrated remarkable therapeutic potential, but its clinical application has outpaced our understanding of how rTMS induces these changes. Knowledge of how rTMS enacts change at the neuronal level may be necessary to optimize therapeutic treatment effects and may be leveraged to improve clinical outcomes in brain medicine.

High-frequency rTMS (≥ 5Hz) is theorized to work through a form of synaptic plasticity called long-term potentiation (LTP), which is mediated by N-methyl-D-aspartate receptors (NMDARs) [1]. 10-Hz magnetic stimulation of mouse hippocampal slices showed changes in hallmark features of LTP such as increased α-amino-3-hydroxy-5-methyl-4-isoxazolepropionic acid receptor (AMPAR) subunit GluA1 expression, dendritic spine size, and NMDAR-dependent post-synaptic currents [2]. Curiously, the same 10-Hz stimulation protocol decreased gamma-aminobutyric acid receptor (GABAR)-mediated inhibitory currents, synaptic expression, and associated scaffolding proteins [3]. Likewise, NMDAR partial agonist, d-cycloserine (DCS), was sufficient to enhance 10-Hz rTMS-mediated facilitation of corticomotor plasticity in humans [4, 5], particularly in those with prior motor learning [6] and without chronic exogenous overexcitation with caffeine which may occlude plasticity [7]. Further, DCS produced apparent occlusion of intracortical facilitation (ICF) and initiated homeostatic depression as revealed via short-interval intracortical inhibition (SICI), both consistent with LTP-like effects [8].

The role of GABARs in faciliatory rTMS protocols has been explored to a lesser extent, though GABA_A_ agonist, diazepam, and GABA_B_ agonist, baclofen, both effectively blocked LTP-like effects of paired associative stimulation (PAS) [9, 10]. These data would speak against a GABAergic reduction mechanism of this excitatory rTMS protocol. Pharmacologic modulation of rTMS protocols has been effectively reviewed elsewhere [11].

rTMS parameters such as train duration and pulse number can produce opposing effects in excitation, limiting short-hand use of “excitatory” and “inhibitory” for respective high-frequency and low-frequency protocols [12–14]. Prior motor physiology studies investigating rTMS mechanisms have focused on parameters of unclear relevance to the FDA-cleared protocol for depression because they differ substantially from the 3000 pulse, 4 second train duration, 26 second intertrain interval, 10-Hz rTMS protocol [15, 16]. For the first time, we present preliminary results on the role of NMDARs or GABARs in this clinically relevant 10-Hz rTMS protocol. We conducted a four-arm crossover study with pharmacologic augmentation of NMDARs and GABARs in the healthy motor cortex measuring plasticity with motor-evoked potentials (MEPs). We hypothesized that 10-Hz rTMS works through both NMDAR-dependent LTP and GABAergic reduction based on foundational animal work. We predicted, therefore, that relative to placebo, 1) NMDAR agonist DCS would enhance MEPs (as observed previously with a 300-pulse 10-Hz protocol), 2) that adding NMDAR antagonist (DCS + dextromethorphan) would block or “knock down” this effect, and that 3) GABAR agonism (lorazepam) would produce increased MEP amplitudes after rTMS, in line with reduced GABA receptors. We also predicted that rTMS effects would peak at 30 minutes after rTMS as observed previously [4].

## Methods

**Figure 1:**
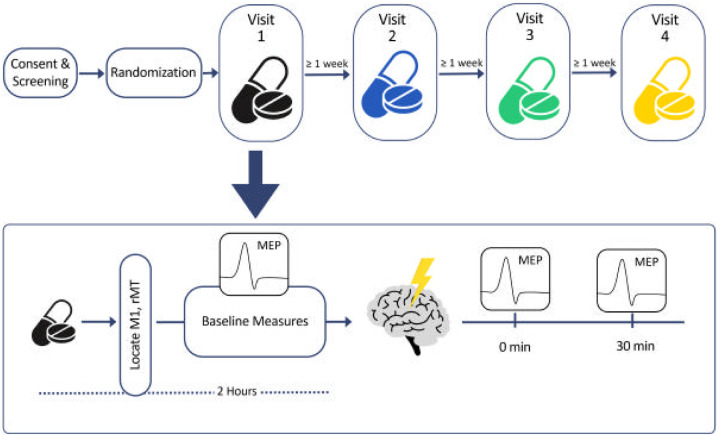
Experimental design. Top: Overview of full experiment with four pill icons representing four drug conditions in randomized order. Bottom: Overview of TMS visit. M1 = motor hotspot, rMT = resting motor threshold, MEP = motor evoked potential. Brain icon = 3000 pulses of 10-Hz repetitive transcranial magnetic stimulation with 4-second train duration and 26-second intertrain interval.

Six healthy participants (four female) aged 21–51 with no brain disorders or current use of psychotropic medications provided informed consent and completed a double-blind randomized study with four crossover drug arms (24 experimental subject visits). The rationale for a 4-arm study was to directly compare the relative contribution of each drug effect on TMS within subjects, as direct comparisons would be limited if each experiment were done separately. All study components were approved by the Butler Hospital Institutional Review Board. An online randomizer was used to generate random sequence of drugs for participants. We excluded individuals with TMS contraindications of safety concerns, such as implanted metal in the skull and elevated seizure risk. In each visit, participants received a single dose of one of the following identical capsules: placebo (sucrose), d-cycloserine (DCS, 100 mg), dextromethorphan (DMO, 150 mg) + d-cycloserine (DCS, 100 mg), or lorazepam (LZP, 2.5 mg). We note that this is a high dose of benzodiazepine, but represents the dose used in prior literature with LZP, as reviewed previously [17]. Both pre-rTMS and post-rTMS MEPs were recorded in the presence of drug to determine the effect of receptor-modulation on 10-Hz rTMS (Fig. 1).

All TMS was done at the motor cortex using the PowerMAG EEG100 power unit and PMD70-pCool Coil (Mag&More, Germany). At each TMS research visit, we obtained resting motor thresholds (rMT), defined as the lowest stimulator intensity to elicit ≥ 5/10 MEPs of peak-to-peak MEP amplitude of at least 50 μV to determine stimulation intensity for each participant. MEPs were then recorded from the right first dorsal interosseous (FDI) muscle with surface electromyography (EMG) electrodes (Cardinal Health, USA) upon stimulating the left motor cortex “hotspot.” Single pulse induced MEPs were collected before and after 10-Hz rTMS at each study visit. Pulses were kept within 0.5 mm of target with neuronavigation performed with a Brainsight 2 System (Rogue Research, Quebec, Canada). Study visit procedures are shown in Fig. 1.

Following drug administration, TMS safety screening questionnaires, and rMT determination (~ 1 hour), we measured baseline motor cortical excitability with 4 sequential bins of 10 single pulses (SP; 40 total, 4–7 seconds jitter) at 120% rMT. 10 MEPs have been previously shown to be sufficient in producing consistent between-session MEP amplitudes in healthy participants [18]. 10-Hz rTMS was administered two hours after drug administration at peak drug bioavailability to the FDI hotspot (3000 pulses, 4 seconds on/26 seconds off) at 80% rMT. 80% rMT was used to minimize seizure risk. Baseline measures were then repeated, with SP MEPs measured immediately (0-min), and 30 minutes (30-min) after 10-Hz rTMS. Tolerability and effectiveness of blind was evaluated after each visit. Participants reported an average rating of 13.9/100 tolerability (100 being extremely painful) and correctly guessed which drug they received 42% of the time with 69% confidence.

All data were analyzed with R software (version 4.2, R Core Team, Vienna, Austria). Within each drug condition, we normalized each rTMS MEP by the average of all 40 baseline MEPs. We used within subject and between-drug analyses to examine the potential differences between each active drug condition relative to placebo. Using repeated measures ANOVA, we tested 30-min post rTMS for effects of drug, time, and drug*time interaction across the four sequential SP bins. We compared each active drug condition with placebo, as well as DCS v. DCS + DMO for a total of four between drug comparisons. As this was a pilot study with *a priori* hypotheses, we did not correct for multiple comparisons.

All data were normalized to baseline grand averages which were calculated by averaging all baseline SPs individually as denominator for each separate bin. Thirty-minute MEPs were our target potentiation sample based on our prior time course findings (Brown, DeVries et al. 2020) and were analyzed using one-way ANOVA with post-hoc Dunnett T3 test to determine potential differences between each drug relative to placebo. As normality conditions in this pilot study were not met by Shapiro-Wilk test, Wilcoxon Signed Rank test was used to assess non-parametric differences between comparisons of interest (i.e., PBO vs DCS, PBO vs LZP, PBO vs DCS + DMO, DCS vs DCS + DMO).

## Results

**Figure 2:**
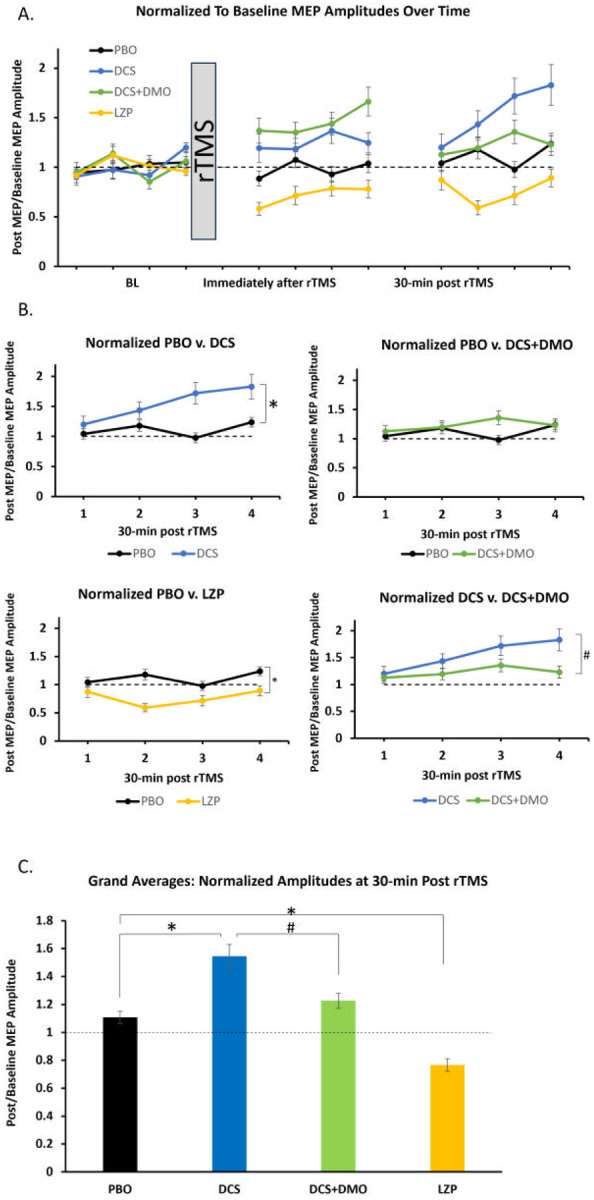
SP time course normalized to baseline (dashed line). (A) Normalized to Baseline MEP Amplitudes Over Time, 0-min post rTMS, and 30-min post rTMS. (B) SP time course at 30-min post rTMS. PBO v. DCS: drug (p < .001), time (p = .001), drug*time (p = .028). PBO v. DCS+DMO: drug (p = .247), time (p = .394), drug*time (p = .068). PBO v. LZP: drug (p < .001), time (p = .030), drug*time (p = .047). DCS v. DCS+DMO: drug (p = .039), time (p < .001), drug*time (p = .129). (C) Grand averages: Normalized Amplitudes at 30-min Post rTMS. One-way ANOVA: F(3, 956) = 29.5, p < .001. Post-hoc (Dunnett T3): PBO v. DCS, p < .001; PBO v. DCS+DMO, p = .429; PBO v. LZP, p < .001; DCS v. DCS+DMO, p = .010. MEP = motor-evoked potential, BL = baseline, PBO = placebo, DCS = d-cycloserine, DMO = dextromethorphan, LZP = lorazepam. * = p < .05 between active drug and placebo, # = p < .05 between DCS and DCS+DMO conditions. Error bars ±1 standard error of mean.

Summarily, Fig. 2A shows data from all drug conditions and all three time points. To test our hypothesis, we analyzed differences of each drug vs. placebo 30-min after rTMS. Consistent with our hypothesis, DCS potentiated rTMS effects compared to placebo (Fig. 2B; F(1, 3) = 13.5, p < .001, η_p_^2^ = .186) with effect of time (F(1, 3) = 5.55, p = .001, η_p_^2^ = .086) and drug*time interaction (F(1, 3) = 3.12, p = .028, η_p_^2^ = .050). Observed power for DCS effect was .950. DCS was compared to DCS + DMO to evaluate specificity of NMDAR effects of DCS and to determine the necessity and sufficiency of NMDAR activity in rTMS facilitation. This comparison revealed significant facilitation of the DCS condition relative to DCS + DMO (F(1, 3) = 4.48, p = .039, η_p_^2^ = .071). Further, DCS + DMO did not differ from placebo (F(1, 3) = 4.48, p = .039, η_p_^2^ = .071), drug*time interaction (F(1, 3) = 1.92, p = .129, η_p_^2^ = .031), but did show an effect of time (F(1, 3) = 5.48, p < .001, η_p_^2^ = .085). Testing for GABAergic reduction, LZP demonstrated significant depression of MEP amplitudes relative to PBO (F(1, 3) = 20.7, p < .001, η_p_^2^ = .260), with an effect of time (F(1, 3) = 3.06, p = .030, η_p_^2^ = .049) and drug*time interaction (F(1, 3) = 2.71, p = .047, η_p_^2^= .044). Observed power for LZP drug effect was .994.

Given non-normality, we analyzed grand averages with active drug conditions vs. placebo at our hypothesized potentiation peak of 30-min after rTMS with Wilcoxon Signed Rank test (Fig. 2C). DCS differed from PBO (Z = −3.74, p < .001), as did DCS from DCS + DMO (Z = −2.755, p < .001) indicating specificity of NMDAR agonism effects by DCS. DCS + DMO did not differ from PBO (Z = − .391, p = .695). In the opposite direction, LZP also differed from PBO in PBO v. LZP (Z = −6.08, p < .001) indicating that GABARs were not removed by rTMS.

## Discussion

While preliminary, these data appear to support our hypothesis that NMDA receptors are both necessary and sufficient for 10-Hz rTMS-induced facilitation, which together, strongly suggest an LTP-like mechanism for 10-Hz rTMS. Contrary to our original hypothesis that 10-Hz works through a combination of LTP and GABAergic reduction, our results did not support a GABAergic reduction induced by 10-Hz rTMS. Our working hypothesis of a combined mechanism was based on original findings from Vlachos and colleagues who found that the same 10-Hz protocol produced both LTP-associated changes [2] and GABAR reduction [3]. One of our objectives was to test whether this finding in mice extended to humans.

Our preliminary results are most notably limited by the small sample size of six subjects across four arms (24 visits total). In context, many of the seminal mechanistic TMS studies have had this challenge in common, and many have borne out over time [11, 17, 19, 20]. Nevertheless, replication with larger samples is needed to draw conclusions. Our study does demonstrate the feasibility of a four-arm crossover study, and the potential to observe differences between drug conditions.

As our ultimate aim is to understand how rTMS produces lasting therapeutic effects at a molecular level, which we consider necessary to optimize rTMS clinical outcomes, we will discuss several important limitations of our study highlighting the way forward to achieve this common goal.

First, although pharmacology in humans allows for molecular manipulation, observations from humans are merely an extrapolation of what occurs with LTP. Such changes include an NMDAR-dependent increase in synaptic transmission, specific increase in synaptic expression of GluA1 receptors (a subtype of AMPA receptor), an increase in spine size from scaffolding proteins and actin cytoskeleton, and involvement proteins like CaMKII and PSD-95 (among many other associated findings) [1]. Therefore, future experiments might include replicating experiments in animals to look for these markers. DCS was recently shown to enhance LTP in rat hippocampal slices using electrical stimulation [21]. What is more interesting, is that a more robust LTP protocol allowed for only a modest increase, whereas a weaker LTP protocol allowed for greater enhancement from DCS. This may explain why DCS occludes iTBS [22, 23] but not in 10Hz rTMS [4, 24, 25] as we’ve suggested previously [26].

Second, TMS-induced plasticity in the human motor cortex may differ from plasticity at the dorsolateral prefrontal cortex – the stimulation site for the treatment of depression. The motor cortex is, by far, the most common site assessed with pharmaco-rTMS plasticity studies due to the convenience of MEPs [11]. However, regional differences in cortical excitability call into question the translatability of corticomotor findings to other cortical regions [27–32].

Third, synaptic plasticity differs between healthy and depressed brains. Decrements in the products of synaptic plasticity (AMPAR gene expression, PSD-95, and synapse numbers) have been observed in post-mortem studies of MDD vs healthy controls [33–35] corresponding with reduced excitability observed in TMS-EEG assessments [36]. Interestingly, post-mortem reductions in NMDAR subunits, NR2A and NR2B, in MDD patients vs healthy controls would account for decreased plasticity, but NR1 subunit levels were unchanged [34]. The NR1 subunit contains the DCS binding site, which may explain why DCS was effective in a clinical trial for depression [37], and why it rescued rTMS-induced excitability in depressed brains near the level of healthy controls [38].

Fourth, even small changes in TMS parameters can produce mitigated or even opposing results [12]. The first study to demonstrate pharmacologic augmentation of 10-Hz rTMS [4] utilized a motor physiology protocol of 300 pulses with 1.5-second train duration and 58.5-second intertrain interval because it produced facilitation over time [13]. However, the same study found that a 5-second train duration with a 55-second intertrain interval produced inhibition. Accordingly, we piloted the 3000-pulse protocol and 4/26 second duty cycle as used clinically to determine whether these effects were similar to the shortened 10-Hz protocol used previously [4, 5]. While our results were similar, we do not know if the 80% resting motor threshold intensity (which we used to reduce seizure risk) equates to the effects of 120% used clinically.

Our original hypothesis was that GABAergic reduction is a part of the 10-Hz rTMS mechanism of action. We used lorazepam, a GABA_A_ agonist, because it has a similar time-to-peak as DCS and DMO (2 hours) allowing for blinded administration across all drugs (taken at same time relative to TMS) (Lexicomp, 2024). Because it has been previously shown to reduce baseline cortical excitability in paired-pulse TMS experiments [39, 40], we reasoned that although baseline MEP amplitude should be lower, amplitudes should be relatively higher after rTMS because there would be fewer GABARs as substrate for the LZP. Additionally, LTP-like effects may also be present, further increasing the MEP amplitudes. However, the observed post-rTMS reduction countered our hypothesis. We aimed to deliver rTMS at the 2-hour mark, when bioavailability was highest. Baseline measures are therefore collected ~ 1 hour after drug administration. As a result, it is possible that brain concentrations of LZP were greater after rTMS (3 to 3.5 hours after ingestion), which would reduce MEPs, and represent a possible confound to our results. By contrast, DCS and DMO have no effect on baseline excitability. This can be tested by giving lorazepam an hour earlier so that peak dose is timed with first assessment rather than rTMS.

This four-arm pilot crossover study is the first evaluation of the role of NMDAR vs. GABAR in rTMS-induced plasticity. It is also the first mechanistic examination of the full 3000 pulses, 4-second train duration, 26-second intertrain interval, 10-Hz protocol, as commonly used in the clinic and FDA-cleared since 2008 [15]. Notable differences are that 80% of resting motor threshold intensity was delivered to the motor cortex, limitations which were addressed above. Finally, it is the first in-human demonstration of a “knock down” effect of the NMDAR-mediated augmentation of 10-Hz rTMS, which has been replicated with motor physiology and a clinical trial [4, 5, 37, 41]. Our objective in combining DCS and DMO into a single capsule was to circumvent a potential floor effect from modest 10-Hz enhancement and to test the specificity of NMDAR-mediated effects of DCS.

Arguably one of the most important innovations in the field of TMS is the finding that DCS combined with iTBS produced more than two-fold clinical improvement in a randomized controlled trial [37]. This is the only clinical trial to date to use this approach, but it represents the vast potential benefits of leverage mechanistic knowledge to augment underlying TMS mechanisms. Neurophysiology studies suggest that other approaches may also be successful [11].
